# An energy efficient design of a multi-layered crossover based 3:8 decoder using quantum-dot cellular automata

**DOI:** 10.1016/j.heliyon.2022.e11643

**Published:** 2022-11-15

**Authors:** Rajasree Das, Md. Shah Alam, Kazi Tanvir Ahmmed

**Affiliations:** Department of Electrical & Electronic Engineering, University of Chittagong, Chittagong-4331, Bangladesh

**Keywords:** Quantum dot cellular automata, Inverter, Majority gate, Logic gate, Decoder

## Abstract

Quantum Dot Cellular Automata (QCA) is advancing as an expectant and ongoing nanotechnology that relies on the behavior of electrons interacting with each other in a quantum cell where a single quantum cell acts like a molecule. This emergent technology promises to deal with the limitations of CMOS technology offering very low power operation with high speed. This paper presents an efficient 3:8 decoder using multilayer crossover technique and successfully implemented by QCA. The proposed decoder is more fault tolerant, having high performance and zero crosstalk due to adopting multilayer crossover technique. A comparative study also shows that the proposed design is less complex, dissipates less power and is more cost effective i.e. almost half of the cost of existing decoder having coplanar type. To validate our proposed design QCA Designer tool has been used.

## 1 Introduction

Following to Moore's prediction, the capacity of the integrated chip will grow exponentially with time. Nanotechnology is a must for future generation Integrated Circuits(IC) development to support 6G communications [1], quantum computing [2], big data analytics [3] and artificial intelligence [4]. In the last few decades, CMOS technology has significantly contributed to this advancement. However, this technology is facing major challenge to operate at nano scale range due to its physical limitations such as short channel effects, doping fluctuations, ultra-thin gate oxides, excess power dissipation and expensive fabrication process [5], [6], [7].

Quantum Dot Cellular Automata (QCA) can solve the physical limitations of CMOS hence drastically increasing the system's performance allowing faster speed, higher integration, higher switching frequency, smaller size and low power consumption. With QCA, nanoscale devices with highly integrated density can be made to operate computational performances at a very high switching speed. The interacting QCA cells have started to be shrunk thereby causing its device density to increase. Also recent researchers have introduced that temperature variations nearby room temperature will hardly cause any effect on QCA operations [8]. Therefore, various digital sequential and integrated circuits like memory [9], flip flops [10], [11], multiplier [12], [13], adders [14], [15], [16], [17], CLB implementation of an FPGA [18], Synchronous Counters [19], Binary-to-Gray Converter [20], multiplexer [21], RAM [22], even parity generators [23] and decoders [24], [25], [26], [27] are designed using QCA implementation.

In modern technology, decoder is playing a vital role among different digital circuits. Due to the increasing applications of decoder circuits in modern technology and the ever shrinking chip size, a novel decoder circuit must be designed at a nano-level using a computer based software system so that we can observe its performance before deploying it into any hardware system. In this view, QCA can be quite advantageous to observe the decoder performance as this software uses quantum cells in the nano-scale range [5].

However, it is observed that 2:4 Decoder circuitry has been designed differently using various gates in a single layer which can cause the rise of complexity in cell optimization [28]. Again for designing a higher level 3:8 Decoder, this complexity increases far more due to the increment of cell intensity and cell wire overlapping within a single layer. Several types of decoder circuits are designed so far using QCA simulation based on its gate structure and connection system [29], [30]. In this study we proposed an optimal decoder circuit designed using multilayer crossover in QCA technology with basic gates. When compared to coplanar crossover, multi-layer crossover has various benefits, including being more fault tolerant, having excellent performance, and provides more flexibility in design [25], [26]. A bridge-like structure is formed to let a wire crossing over with another wire in crossover technique so that we may overcome the crosstalk between the interconnects and make the circuit more fault tolerant. We have designed a multilayer crossover based 3:8 decoder using simple logic gates in this paper. The aim is to design a decoder with less complexity, maximum utilization of the sample area and minimal power dissipation. The number of majority gates, crossover and the inverters plays a significant role to calculate the cost function which eventually measures the complexity of the decoder circuit. By calculating the cost function, we have found that our proposed decoder costs almost half of the present decoders using coplanar technique.

## 2 Quantum dot cellular automata

### 2.1 QCA cell architecture

**Figure 1:**
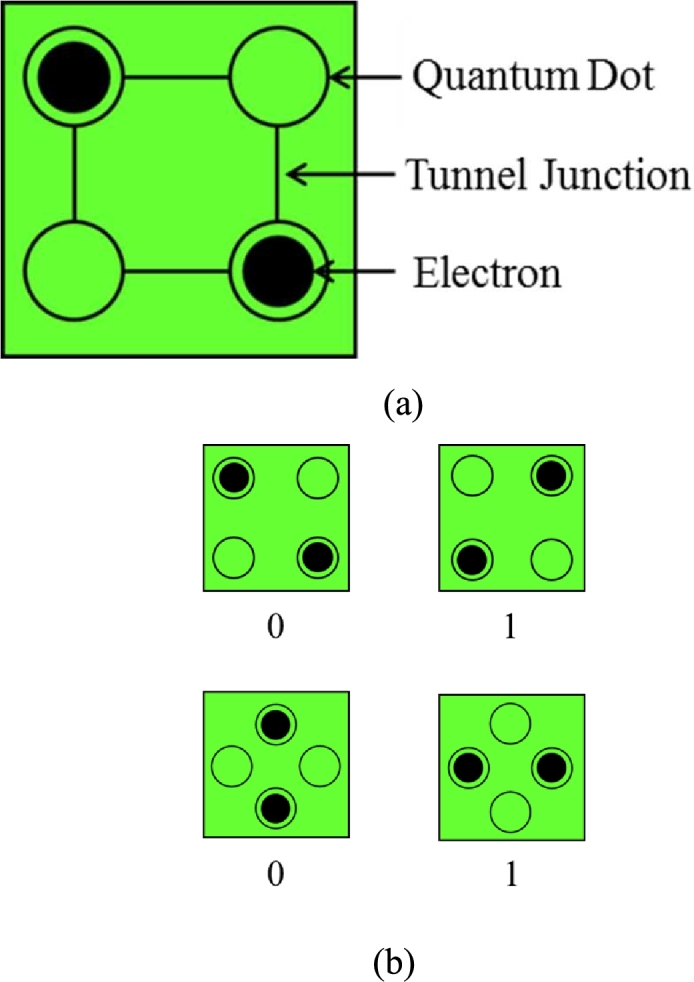
(a) QCA Cell [32] (b) Charge Polarization of “-1” binary 0 and “+1” binary 1 [34].

A QCA cell is square in shape containing four quantum dots at each corner of the cell Fig. 1 (a) [31], [32]. Two excess electrons are injected into the QCA cell which reside inside any two dots to make the cell polarized [33].

As the cell is polarized with two excess electrons, these two electrons always tend to stay as far as possible due to their electrostatic repulsion force, also known as Columbic Repulsion Force [32]. Due to this repulsion force, two electrons are commonly arranged diagonally [35]. These two electrons have access to tunnel within their adjacent dots whenever any input signal is encountered [36]. Therefore, two types of cell polarization are obtained where Polarization = “+1” refers to binary logic “1” and Polarization = “-1” refers to binary logic “0” simultaneously shown in Fig. 1 (b) [34], [37]. The QCA cell polarization can be expressed as the below equation [15], [24].

(1) $$P=\frac{\left(\rho_{1}+\rho_{3})-(\rho_{2}+\rho_{4}\right)}{\rho_{1}+\rho_{2}+\rho_{3}+\rho_{4}}$$

Here two different polarities are created based on how the electrons are located in the cell. According to Eq. (1), placing the electrons in p1, p3, and p2, p4 locations results in polarities *P* = ‘+1’ and *P* = ‘-1’ respectively: these are equivalent to binary values, ‘1’ and ‘0’.

### 2.2 QCA cell wire

**Figure 2:**
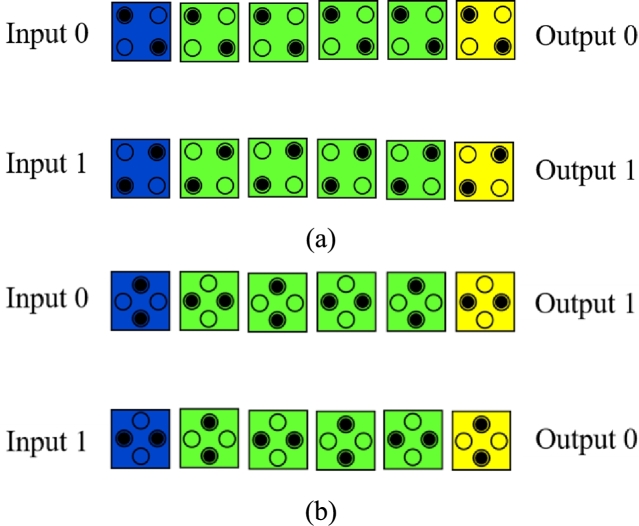
(a) “90°” QCA wire (b) “45°” QCA wire [40].

We can design a QCA Cell Wire by arranging two or more QCA cells alongside to form a linear array, known as binary array or binary wire [38]. There are two types of QCA wire arrangements, one is “90°” QCA wire and the other is “45°” QCA wire shown in Fig. 2 (a, b) [39], [40].

In “90°” QCA wire, the polarization remains similar for all the cells within the entire length wire. Therefore, the output always remains same as the input [33] but in “45°” QCA wire, the binary signal of one cell reverses to its complement value as it propagates through one after another cell.

### 2.3 Majority gate

**Figure 3:**
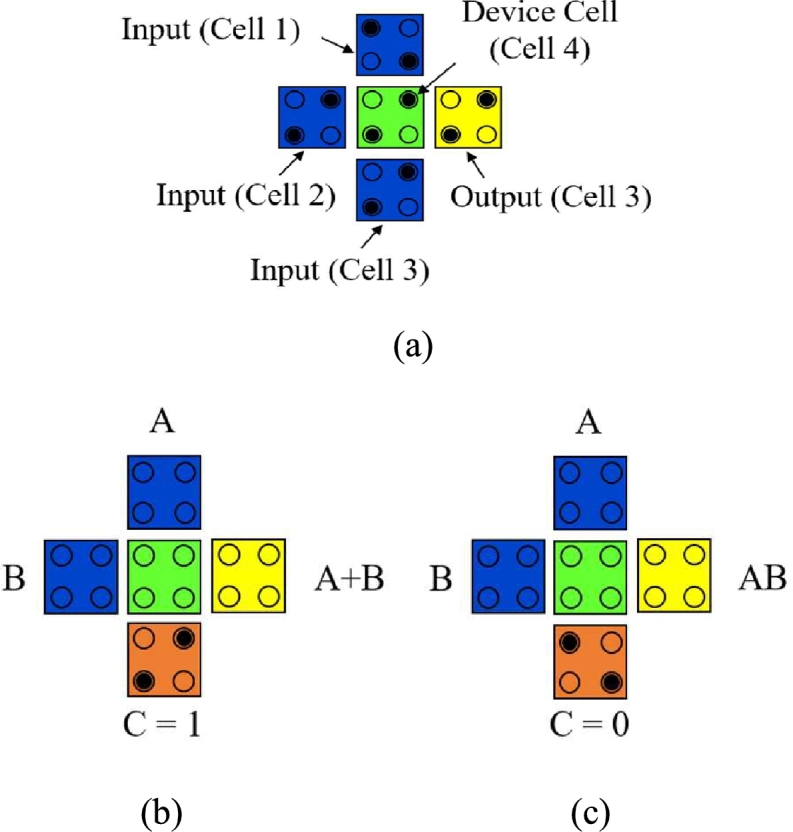
(a) Three input Majority Gate (b) 2-input OR Gate (c) 2-input AND Gate [41].

Basically, a Majority Gate builds up consisting of five cells where three cells operate as input cells, one as the output cell and the other as a middle cell. Fig. 3 shows the 3-input majority gates [37], [41], [42].

The output of the three input (A, B, C) Majority Gate follows a Boolean logical function [43], which can be expressed as [11], [41],

(2) M (A, B, C)=AB+BC+CA

This middle cell may operate as a 2-input OR Gate when P=+1(logic1) as shown in equation (3). Alternatively, to operate as 2-input AND Gate, polarization P=−1(logic0) is applied to its 3^rd^ input cell as shown in equation (4).

(3) M (A, B, 1)=AB+B.1+1.A=A+B

(4) M (A, B, 0)=AB+B.0+0.A=AB

### 2.4 Inverter gate

When a polarized cell is set alongside with another polarized cell, the electrons of one cell are repelled each other by the electrons of the other cell due to columbic interaction. Thus the state propagating through the wire reverses sequentially as passing from one cell to the next cell. This property leads to an opportunity to build up an Inverter gate [44].

**Figure 4:**
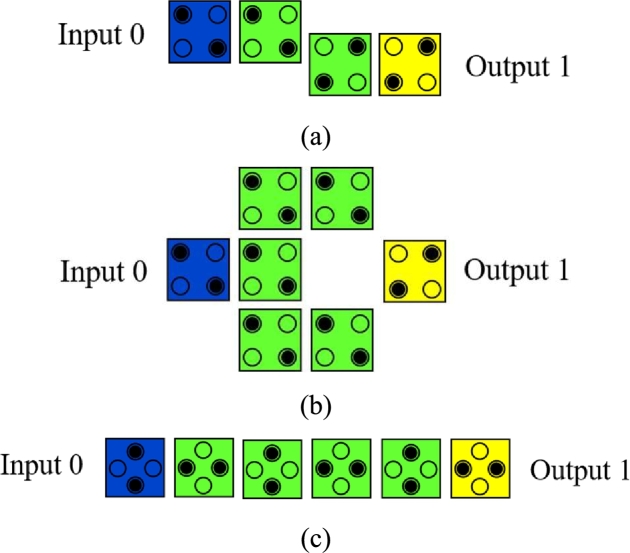
(a) 2-cell Inverter Gate [45] (b) QCA Inverter Gate [45] (c) 45^∘^ rotated Inverter chain [46].

A QCA Inverter gate can be designed in three ways and depicted in Fig. 4 (a, b and c). These are: (a) By displacing two cells with respect to each other, (b) By placing the cells diagonally to each other and (c) By using 45^∘^ oriented cells [5], [34], [43], [45], [46], [47].

### 2.5 QCA clocking

**Figure 5:**
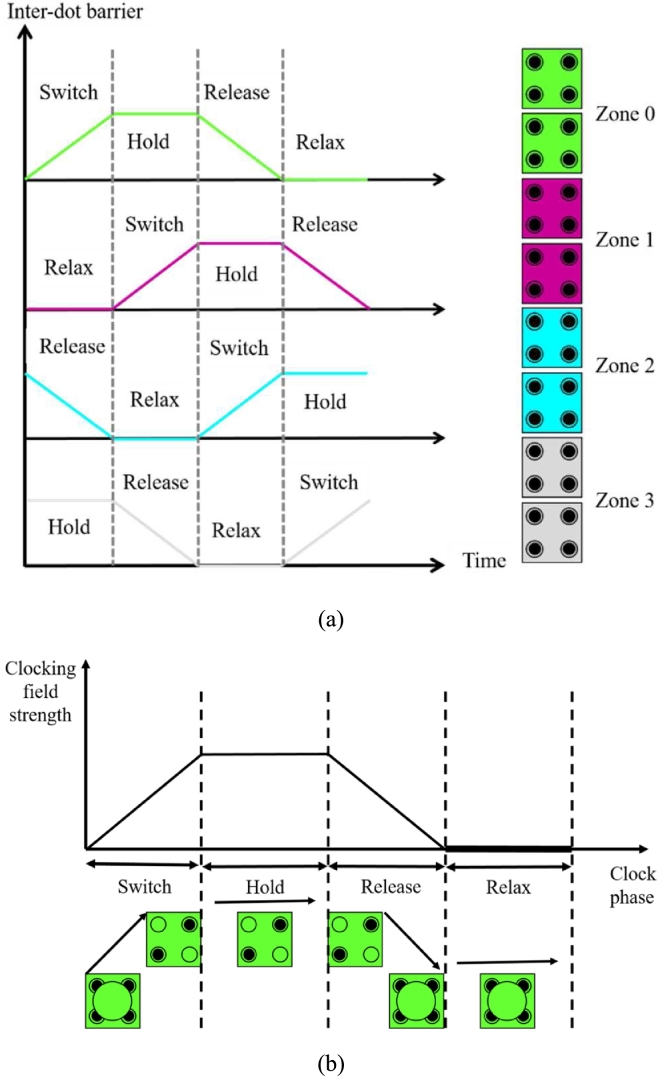
(a) Four clock phases [49] (b) QCA clock behavior showing four phase shifts [53].

In QCA designing, signal information is passed from one cell to the next following the clocking [46]. There are four clock zones named as Clock 0, Clock 1, Clock 2, Clock 3 where each of these clock zones is set at a 90°phase shift with each other respectively [48]. In a clock zone all the phases are repeated sequentially after completing other phases [Fig. 5 (a)] [41], [49], [50], [51]. In QCA design, Green represents Clock 0, Violet represents Clock 1, Blue represents Clock 2 and off white represents Clock 3 respectively [49]. There are four clock phases in any clock zone termed as **Switch**, **Hold**, **Release** and **Relax** shown in Fig. 5 (b) [52], [53].

In **Switch** phase, the potential barrier between two adjacent cells starts to increase causing the tunneling to decrease under the effect of the adjacent cell. Thus, the cell gets polarized. In **Hold** phase, the potential barrier reaches to the highest value and the tunneling of electrons is stopped. Since the electrons cannot switch, they hold their polarity. In **Release** phase, the potential barrier starts to decrease and the tunneling gets started. Thus the cells tend to lose their polarity. In **Relax** phase, the potential barrier reaches to the lowest value and the tunneling of electrons becomes fully opened. Thus, there is no polarization under the influence of its adjacent cell [7], [52], [54]. All the above-mentioned clock phases along with their polarization states are summarized in Table 1.

**Table 1:** Operation of QCA clock Phases [11], [56].

Clock phases	Potential barrier	Polarization states of the cells
Switch	Low to high	Polarized
Hold	High	Polarized
Release	High to low	Un-polarized
Relax	Low	Un-polarized

### 2.6 QCA crossover

**Figure 6:**
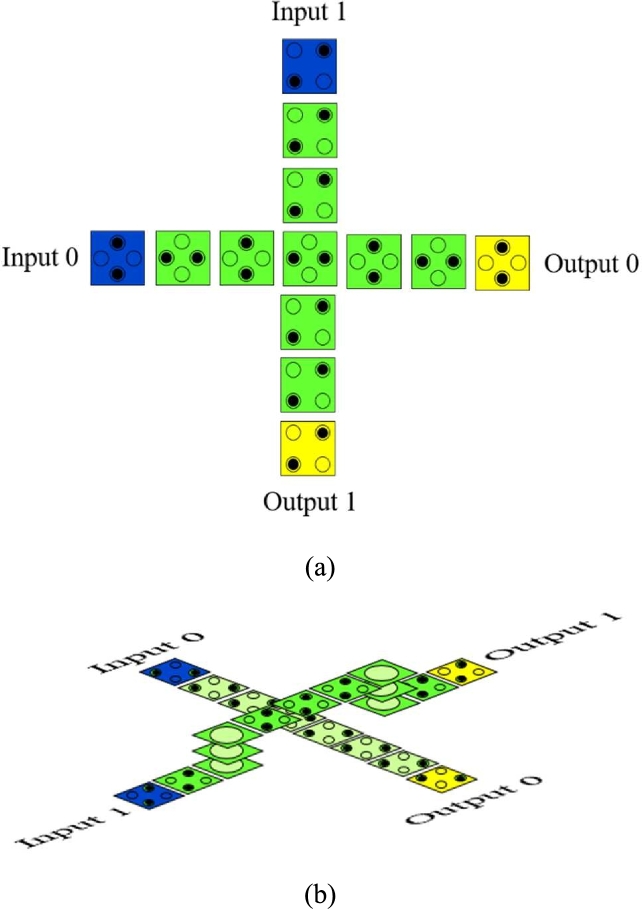
(a) Coplanar Crossover (b) Multi-layer Crossover [56].

In QCA two types of wire crossings are used: the coplanar crossover and the multi-layer crossover [Fig. 6] [55], [56]. Coplanar crossover uses two wires of different orientation where one wire is a binary wire (90°) and the other wire is an inverted chain wire (45°) [57]. There the wires are designed perpendicular with each other [40]. This crossover can be done using only a single layer [36].

Multi-layer crossover uses three layers (Main layer, via layer and Crossover layer) to interconnect two cell wires [38], [58]. In multi-layer crossover, a bridge-like structure is formed to let a wire crossing over with another wire [59]. Thus signals can easily pass through the wires as they are vertically interconnected [60]. Multi-layer crossover provides more authentic information compared to the coplanar crossover as it does not vary with any surrounding fluctuations [40]. Multi-layer crossover occupies several advantages compared with coplanar crossover like being more fault tolerant, having high performance and zero crosstalk between the interconnections [61]. Thus, we have chosen multilayer crossover technique to design 3:8 decoders.

## 3 Decoder

**Figure 7:**
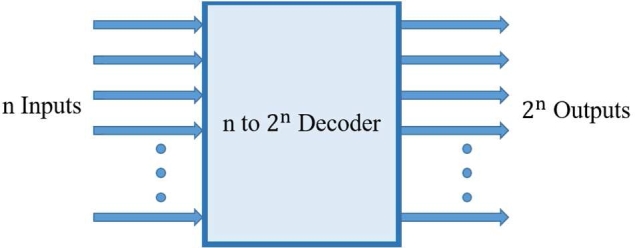
Basic Decoder circuit block diagram [62].

A Decoder plays an undeniable role in logic circuits for all data processing and communication networks. It is a combinational logic circuit that converts binary information from n inputs into $2^{n}$ unique outputs [Fig. 7] [62]. One of these outputs will be active high based on the combination of the inputs present which means decoder detects a particular code.

We can design the “2:4 Decoder” by using a logic circuit that consists of 2 input lines, and giving 4 corresponding output lines. In QCA design, four three-input majority voter gates are used where one of each majority voter gate inputs is always fixed on logical 0 with a polarization, P = -1, which results in the function of the majority voter gate as the AND gate. Majority functions of the four outputs of the circuit are:

F0 = ${\mathrm{MV}}_{1}$(A', B', 0)

F1 = ${\mathrm{MV}}_{2}$(A', B, 0)

F2 = ${\mathrm{MV}}_{3}$(A, B', 0)

F3 = ${\mathrm{MV}}_{4}$(A, B, 0)

### 3.1 3:8 Decoder

**Figure 8:**
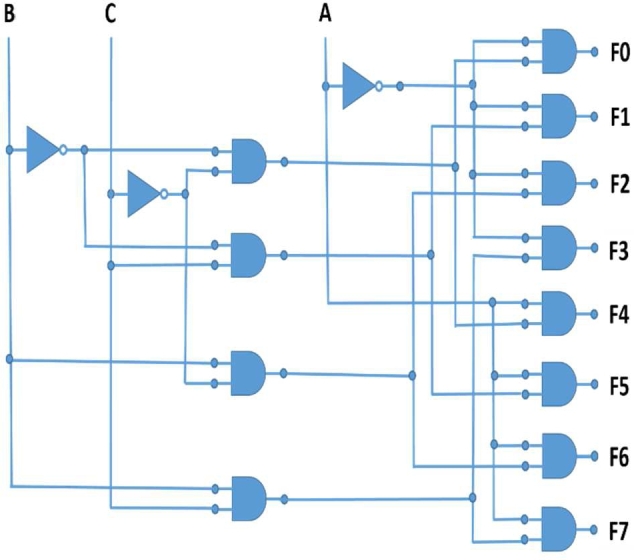
3:8 Decoder Circuit Diagram [36].

We have designed the “3:8 Decoder” by using a logic circuit that consists of 3 input lines and giving 8 corresponding output lines [Fig. 8]. Table 2 represents the truth table of 3:8 decoder.

**Table 2:** 3:8 Decoder Truth Table [36].

Input			Output							
A	B	C	F0	F1	F2	F3	F4	F5	F6	F7
			A'	A'	A'	A'	A	A	A	A
			B'	B'	B	B	B'	B'	B	B
			C'	C	C'	C	C'	C	C'	C
0	0	0	1	0	0	0	0	0	0	0
0	0	1	0	1	0	0	0	0	0	0
0	1	0	0	0	1	0	0	0	0	0
0	1	1	0	0	0	1	0	0	0	0
1	0	0	0	0	0	0	1	0	0	0
1	0	1	0	0	0	0	0	1	0	0
1	1	0	0	0	0	0	0	0	1	0
1	1	1	0	0	0	0	0	0	0	1

In QCA design, 12 three-input majority voter gates are used where one of each majority voter gate inputs is always fixed on logical 0 with a polarization, P = -1, which results in the function of the majority voter gate as the AND gate. Among these, the eight majority voter gate gives the corresponding outputs. Majority functions of the eight outputs of the circuit are:

F0 = ${\mathrm{MV}}_{1}$(MV(B'.C'), A', 0)

F1 = ${\mathrm{MV}}_{2}$(MV(B'.C), A', 0)

F2 = ${\mathrm{MV}}_{3}$(MV(B.C'), A', 0)

F3 = ${\mathrm{MV}}_{4}$(MV(B.C), A', 0)

F4 = ${\mathrm{MV}}_{5}$(MV(B'.C'), A, 0)

F5 = ${\mathrm{MV}}_{6}$(MV(B'.C), A, 0)

F6 = ${\mathrm{MV}}_{7}$(MV(B.C'), A, 0)

F7 = ${\mathrm{MV}}_{8}$(MV(B.C), A, 0)

## 4 Proposed design

**Figure 9:**
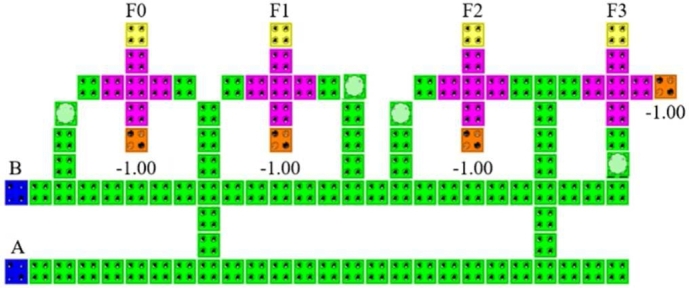
QCA layout for 2:4 Decoder.

We first designed a multilayered based 2:4 decoder (Fig. 9) where the inputs are A and B. Input A and B are logically combined using AND gate giving four corresponding outputs which are obtained from the four majority gates in the main cell layer. The corresponding outputs are F0, F1, F2 and F3, which are found in the main cell layer as all the four majority voter gates are designed in the main cell layer.

**Figure 10:**
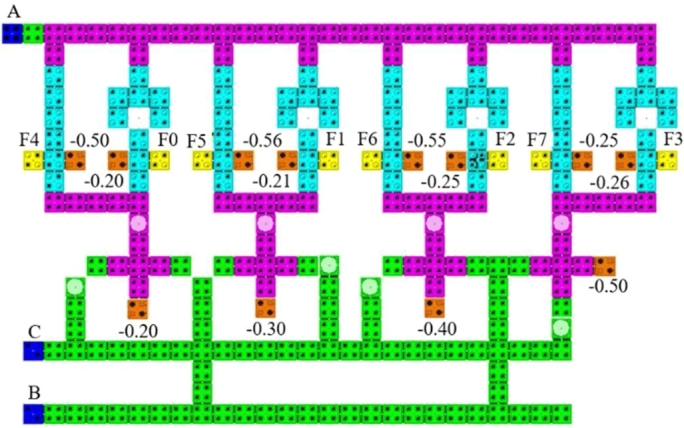
Proposed QCA layout for 3:8 Decoder using 2:4 Decoder.

Using the layout of 2:4 Decoder, we have designed the layout of a multilayered based 3:8 decoder where the inputs are A, B and C (Fig. 10). Input A and C are designed in the upper layer and input B is designed in the main cell layer. At first, input B and C are logically combined using AND gate giving four corresponding outputs which are obtained from the lower four majority gates in the main cell layer. Then, These four majority gate outputs are again logically combined with input A and inverter A using AND gate giving eight corresponding outputs F0, F1, F2, F3, F4, F5, F6 and F7, which are found from the upper eight majority gates as these gates are designed in the upper layer.

## 5 Result analysis

**Figure 11:**
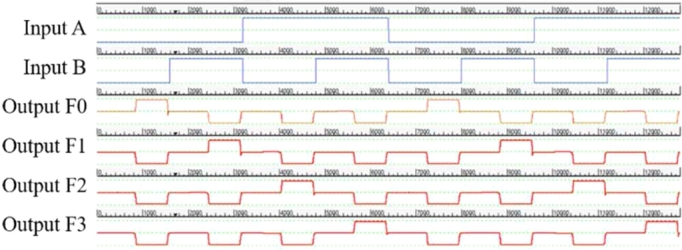
The simulation result of 2:4 Decoder.

**Figure 12:**
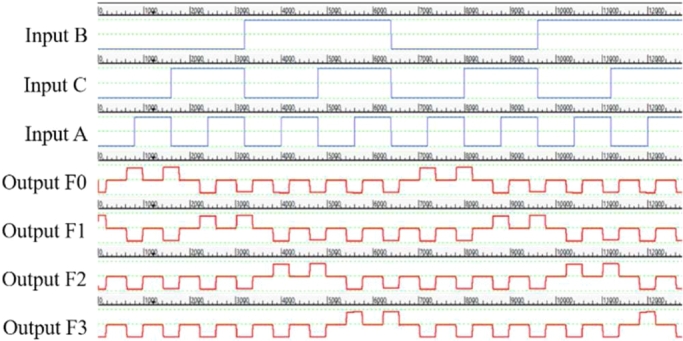
The simulation result showing output F0, F1, F2 and F3 of 3:8 Decoder.

**Figure 13:**
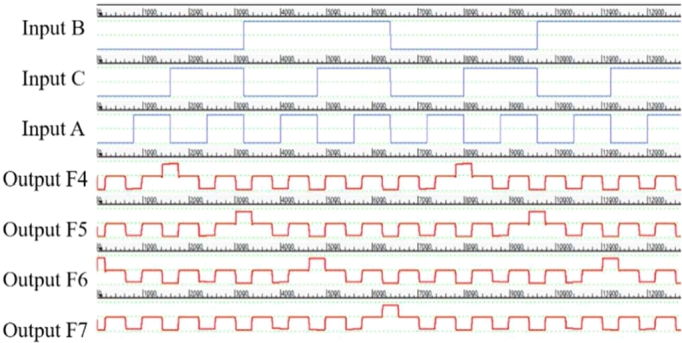
The simulation result showing output F4, F5, F6 and F7 of 3:8 Decoder.

The design, simulation and validation of the proposed decoder circuits are done using QCADesigner 2.0.3 software. First we have observed the simulation results obtained from the multilayered crossover based 2:4 decoder in Fig. 11. Then, we observed the simulation results obtained from the multilayered crossover based 3:8 decoder. To avoid complexity, the simulation results are shown in two steps [Fig. 12 and Fig. 13].

All the design specifications of the 3:8 decoder is tabulated in Table 3 and these parameters set up the simulation engine into Bistable Approximation. The bistable approximation engine calculates state of a single cell using a time-independent approach with kink energy formula that calculates cost of two cells having opposite polarizations, so simulation time in this engine is reduced.

**Table 3:** Parameters used in Bi-stable Approximation [10], [12], [13], [14].

Parameters	Values
Number of samples	500000
Cell Size	18 nm × 18 nm
Radius of Effect	65 nm
Cell Separation	2 nm
Relative Permittivity	12.9
Dot Diameter	5 nm
Clock Amplitude Factor	2
Convergence Tolerance	0.001
Clock High	9.8 e-22 J
Clock Low	3.8 e-23 J
Layer Separation	11.5 nm
Maximum iteration per sample	100

### 5.1 Area calculation

As each quantum cell is square in shape with the sidelength 18 nm, the area 18 nm × 18 nm. In addition, there is a 2 nm gap between the edges of two neighboring cells. Therefore, the occupied area of a QCA sample layout can be calculated as follows [28]:

The sample Area = (The highest cell number in vertical direction) × (The highest cell number in horizontal direction) × 400 ${\text{nm}}^{2}$.

Using the equation, it is calculated that the sample area is 243200 ${\text{nm}}^{2}$ with 32 cells in the vertical lining and 19 cells in the horizontal lining. In the proposed multilayer crossover based design, there are total 263 cells. Out of these only 245 cells have approached the net area which is about 79380 ${\text{nm}}^{2}$. This cell area is about 32.64% out of the total sample area.

### 5.2 Cost function

To measure the complexity of a circuit, it is important to calculate its cost function. The number of logic gates and crossovers have a high impact on calculating the cost function. Because majority gates affect irreversible power dissipation and crossovers are subjected to fabrication difficulty. Moreover, delay should be taken under consideration due to its impact on the performance measurement. A widely acknowledged equation for calculating the cost function of QCA circuits was formerly introduced by Liu and others as follows [63]:

(5) $${\mathrm{QCA}}_{\mathrm{cost}}=(M^{k}+I+C^{l})\times T^{p},\;1\leq k,l,p$$

Here, M = number of majority gates.

  I = number of inverters.

  C = number of crossovers.

  T = delay of the circuit.

  k, l, and p = exponential weightings for

  majority gate count, crossover

  count and delay respectively.

**Figure 14:**
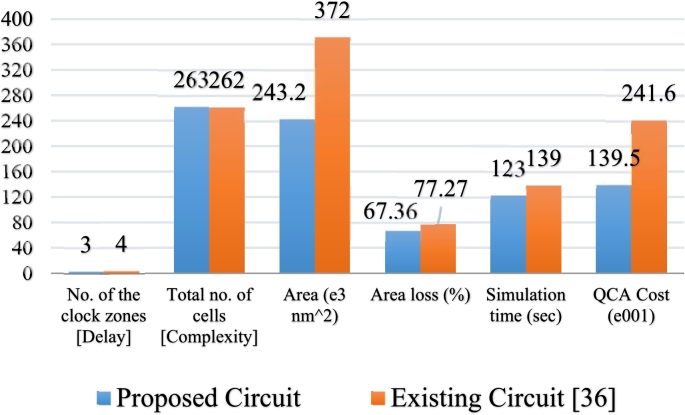
A comparative analysis of different decoders.

As majority gates affect the complexity and energy dissipation, a double weight is counted for the M parameter, that is, k = 2. Similarly, l = 2 for the C parameter, as crossovers affect the complexity and fabrication difficulty. Again, the delay of the circuit affects the clock speed and polarization, thus the weighting used for this is p = 2 and a constant weighting 1 is settled down for I, as inverters only affect the complexity of the QCA circuits. A comparative study is shown in Fig. 14.

$${\mathrm{QCA}}_{\mathrm{cost}}=(12^{2}+7+2^{2})\times 3^{2}=1395$$

It is far less than the coplanar one [36], where the ${\mathrm{QCA}}_{\mathrm{cost}}$ is 2416. This difference exhibits that the proposed design is comparatively less complex and more cost effective than the previous study.

### 5.3 Power dissipation

Each QCA cell causes a uniform power dissipation. Through the process occupied in one clock phase, the power dissipation of the complete circuit is depicted by calculating the power dissipation estimation of all majority gates as well as inverters [64], [65], [66], [67], [68], [69] and memory devices [70], [71], [72], We analyzed the power dissipation of the proposed QCA layout from the temperature 1K to 10K in a discrete tunneling energy in the forms of total energy dissipation per cycle and compared it with the existing circuit [36].

**Figure 15:**
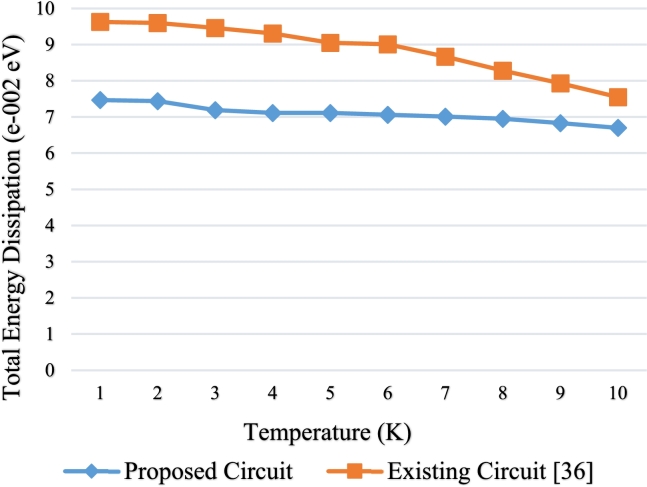
Total energy dissipation vs temperature.

All the values are calculated in QCA Designer-E Version 2.2 using the simulation engine termed as Coherence Vector (w / Energy). A comparative view is shown graphically about total energy dissipation per cycle for the decoder circuits in Fig. 15. These energy dissipation comparison shows the 3:8 decoder dissipates less power when it is designed using multilayer crossover rather than the coplanar one as shown in Fig. 15. It is observed that, the total energy dissipation for our proposed design is less than 1 to 2 e-0002 eV over 1 to 10 K temperature range. This low dissipation ensures high conductivity and low resistivity. This design is highly applicable for semiconductor QCA implemention, as the performance of semiconductive materials increases with the increasing temperature.

Table 4 shows the comparison between our proposed 3:8 decoder with the 2:4 decoders [25], [26]. The advantage of the proposed design is that the output can be achieved just after 3 clock cycles. Ours one of the finest achievements is that we have successfully designed the 3:8 decoder circuit within almost the same area occupied by the 2:4 decoder circuits with reduced power dissipation. Furthermore, our study confirms that the proposed design is cost-effective i.e., almost half of the cost of the previous design [36].

**Table 4:** Comparison among proposed 3 to 8 decoder over some reported 2 to 4 decoder circuits.

Layer type	Majority Voters (MV)	Clock cycle delay	Active area (μm^2^)	Reference
Coplanar (Single)	5 input	3	0.22	[25]
Coplanar (Single)	3 input	6	0.25	[26]
Multilayer crossover	3 input	3	0.28	Ours

## 6 Conclusion

This paper presents an energy efficient 3:8 decoder utilizing the multilayer crossover structure. Over a temperature range of 1 to 10 K, the overall energy dissipation for our suggested design is less than 1 to 2 e-0002 eV. We have successfully designed the 3:8 decoder circuit within almost the same area occupied by the 2:4 decoder circuits with reduced power dissipation and from the proposed decoder the output can be achieved only after 3 clock phases. Our study confirms that, the proposed design is cost effective i.e., almost half of the cost of the previous design. Following this methodology, in the future we can design higher level n:$2^{n}$ decoder (n > 3) for high speed computing and high density applications.

## Declarations

### Author contribution statement

Rajasree Das: Conceived and designed the study; performed the simulation and the acquisition of data; wrote the paper. Md. Shah Alam: Conceived and designed the study; performed the simulation and the acquisition of data; wrote the paper. Kazi Tanvir Ahmmed: Analyzed and interpreted the data; wrote the paper.

### Funding statement

This work was supported by Research and publication cell, 10.13039/100016208University of Chittagong (202/2020).

### Data availability statement

Data included in article/supp. material/referenced in article.

### Declaration of interests statement

The authors declare no conflict of interest.

### Additional information

No additional information is available for this paper.
